# Proteomic and metabolomic analyses reveal metabolic responses to 3-hydroxypropionic acid synthesized internally in cyanobacterium *Synechocystis* sp. PCC 6803

**DOI:** 10.1186/s13068-016-0627-6

**Published:** 2016-10-06

**Authors:** Yunpeng Wang, Lei Chen, Weiwen Zhang

**Affiliations:** 1 Laboratory of Synthetic Microbiology, School of Chemical Engineering and Technology, Tianjin University, Tianjin, 300072 People’s Republic of China; 2 Key Laboratory of Systems Bioengineering (Ministry of Education), Tianjin University, Tianjin, People’s Republic of China; 3 SynBio Research Platform, Collaborative Innovation Center of Chemical Science and Engineering (Tianjin), Tianjin, People’s Republic of China

**Keywords:** 3-HP, Biosynthesis, Response, Proteomics, Metabolomics, *Synechocystis*

## Abstract

**Background:**

3-hydroxypropionic acid (3-HP) is an important platform chemical with a wide range of applications. In our previous study, the biosynthetic pathway of 3-HP was constructed and optimized in cyanobacterium *Synechocystis* sp. PCC 6803, which led to 3-HP production directly from CO_2_ at a level of 837.18 mg L^−1^ (348.8 mg/g dry cell weight). As the production and accumulation of 3-HP in cells affect cellular metabolism, a better understanding of cellular responses to 3-HP synthesized internally in *Synechocystis* will be important for further increasing 3-HP productivity in cyanobacterial chassis.

**Results:**

Using a engineered 3-HP-producing SM strain, in this study, the cellular responses to 3-HP internally produced were first determined using a quantitative iTRAQ-LC–MS/MS proteomics approach and a LC–MS-based targeted metabolomics. A total of 2264 unique proteins were identified, which represented about 63 % of all predicted protein in *Synechocystis* in the proteomic analysis; meanwhile intracellular abundance of 24 key metabolites was determined by a comparative metabolomic analysis of the 3-HP-producing strain SM and wild type. Among all identified proteins, 204 proteins were found up-regulated and 123 proteins were found down-regulated, respectively. The proteins related to oxidative phosphorylation, photosynthesis, ribosome, central carbon metabolism, two-component systems and ABC-type transporters were up-regulated, along with the abundance of 14 metabolites related to central metabolism. The results suggested that the supply of ATP and NADPH was increased significantly, and the precursor malonyl-CoA and acetyl-CoA may also be supplemented when 3-HP was produced at a high level in *Synechocystis*. Confirmation of proteomic and metabolomic results with RT-qPCR and gene-overexpression strains of selected genes was also conducted, and the overexpression of three transporter genes putatively involved in cobalt/nickel, manganese and phosphate transporting (i.e., *sll0385*, *sll1598* and *sll0679*) could lead to an increased 3-HP production in *Synechocystis*.

**Conclusions:**

The integrative analysis of up-regulated proteome and metabolome data showed that to ensure the high-efficient production of 3-HP and the normal growth of *Synechocystis*, multiple aspects of cells metabolism including energy, reducing power supply, central carbon metabolism, the stress responses and protein synthesis were enhanced in *Synechocystis*. The study provides an important basis for further engineering cyanobacteria for high 3-HP production.

**Electronic supplementary material:**

The online version of this article (doi:10.1186/s13068-016-0627-6) contains supplementary material, which is available to authorized users.

## Background

3-hydroxypropionic acid (3-HP) is a non-chiral carboxylic acid which contained a hydroxyl group on its third carbon atom, and has a high potential as a platform compound to produce many other chemicals, such as 1,3-propanediol [1]. In 2010, 3-HP was ranked as one of the top value-added chemicals produced from biomass by US Department of Energy [2]. In addition to chemical synthesis [3], a range of microorganisms, such as *Escherichia coli*, *Klebsiella pneumoniae* and *Pseudomonas denitrificans*, have been engineered for biological production of 3-HP using glycerol [4] or glucose [5] as the carbon sources. To address the need to develop renewable processes for producing chemicals, which will eventually allow substitution of fossil fuels or chemicals, synthetic biology efforts of using engineered photosynthetic microbes to produce chemicals directly using CO_2_ and solar energy have been undergoing in recent years. In our previous study, malonyl-CoA reductase coding gene (*mcr*) of *Chloroflexus aurantiacus* was cloned and introduced into cyanobacterium *Synechocystis* sp. PCC 6803 (hereafter *Synechocystis*) to construct the 3-HP biosynthetic pathway directly utilizing CO_2_ [6]. After several steps of system optimization, including expression increase of *mcr* gene using different promoters, improved supply of precursor malonyl-CoA and NADPH, a production of 837.18 mg/L 3-HP directly from CO_2_ was achieved in the engineered *Synechocystis* after 6-day cultivation [6]. However, when compared with engineered *E. coli* systems, the productivity in *Synechocystis* is still lower, and further efforts to optimize the production system from both pathway and chassis aspects are necessary.

In our previous study, the 3-HP production reached 688 mg L^−1^ in strain SM after enhancement of *mcr* gene expression with high-capability promoter *P*_*cpc560*_. However, in the promoter *P*_*cpc560*_ background, the further supply of malonyl-CoA and NADPH was not significant to the improvement of 3-HP production [6]. In an early study, Vu et al. [7] studied the capabilities of *Synechococcus* PCC 7002 as a chassis for producing several native and nonnative compounds [7]. Although computational experiments indicated that the target chemicals production could be improved through single deletions in central metabolism, the production was not coupled to growth [8]. In addition, the computational analysis showed that many knockouts (i.e., typically 9–10 deletions) were needed to establish growth-coupled mutants [7, 9], suggesting that a global-level metabolic change was typically associated with the production of non-native products in cells. In the case of 3-HP, as it is fully expected that its high production will affect cellular metabolism of *Synechocystis*, a better understanding of these metabolic changes in the 3-HP-producing strains at a systematic level will be valuable for the improvement of 3-HP production.

Metabolic responses to target products in cyanobacteria have recently been investigated through transcriptomics and proteomics studies. However, most of these studies were carried out through the extracellular addition of an end product, e.g., ethanol and butanol, to batch cultures of cyanobacteria, particularly *Synechocystis* [4]. Significant stress responses were reported upon addition of these end products, including up-regulation of heat shock proteins, modification of the cell membrane and cell mobility, as well as induction of the oxidative stress response [4]. Meanwhile, as the effects on cells caused by products produced intracellularly may be different from that induced by exogenously added products, it is necessary to define the metabolic responses of cyanobacterial cells to non-native products at a molecular level. To address the need, a transcriptomic study of prolonged ethanol production in *Synechocystis*, yielding a final level of 4.7 g/L ethanol (i.e., 2.5-fold less than the concentration of ethanol used in [10, 11] to stress the cells), was recently conducted and the results showed that the product formation caused only minor changes at the level of gene expression [12]. Only three mRNAs were found differentially regulated when microarray analyses were performed at day 4, 7, 11 and 18 of the experiment. In addition to up-regulated *adhA* (*slr1192*) gene, expression of *cpcB* (*sll1577*) and *rps8* (*sll1809*) were also down- and up-regulated, respectively, suggesting ethanol production may affect photosynthesis and ribosome in *Synechocystis* [12]. In a very similar study, a proteomic analysis of an ethanol-producing *Synechocystis* strain revealed that the ethanol production resulted in an increase of the overall rate of carbon fixation, and up-regulated a set of proteins involved in the carbon concentrating mechanism, CO_2_-fixation, and the Calvin cycle [13]. Proteomics analysis of lactate-producing *Synechocystis* strain revealed that lactate production broke the balance of the intracellular NADH/NAD^+^ ratio and also affected the photosynthesis [13]. In the cyanobacterial strain over-producing polyhydroxybutyrate (PHB), measurement of the intracellular levels of acetoacetyl-CoA, acetyl-CoA and 3-hydroxybutyryl-CoA (3-HB-CoA), showed that these products were either absent or at markedly low levels [14], suggesting significant metabolic changes upon PHB overproduction. Although work related to optimization of cyanobacterial metabolism for producing non-native chemicals has just recently started, these results have demonstrated that the approach could result in significant improvements in rational strain designs [15].

So far no study on metabolic responses to 3-HP synthesized internally has been reported. Using the 3-HP-producing strain we constructed previously [6], in this study, metabolic responses of *Synechocystis* to 3-HP synthesized internally were determined using an integrated proteomic and metabolomic approach. The results showed that metabolism related to energy, reducing power, central carbon metabolism, protein synthesis, cofactors and amino acid metabolism and stress response mechanism were differentially regulated in the 3-HP-producing strain. The study provides a valuable proteomic and metabolomic view of cellular changes in the 3-HP-producing cell factory and the information could be useful for further engineering the cyanobacteria for high 3-HP production.

## Results and discussion

### 3-HP production in engineered SM strain

**Fig. 1:**
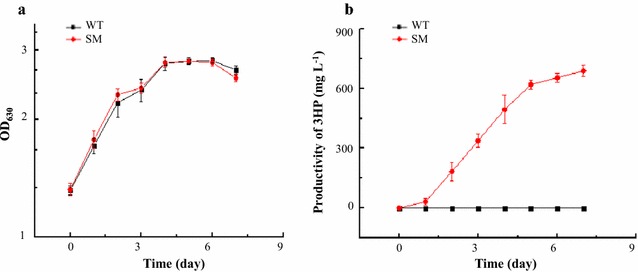
Cell growth and 3-HP production of the WT and the engineered *Synechocystis* SM strains. **a** Growth of the WT and the engineered *Synechocystis* SM strain; **b** 3-HP production by the WT and the engineered *Synechocystis* SM strain

To determine the metabolic responses of *Synechocystis* to 3-HP production, the 3-HP-producing *Synechocystis* strain SM engineered previously [6] and wild type (WT) *Synechocystis* were selected for a comparative analysis. The SM strain expressed malonyl-CoA reductase coding gene (*mcr*) from *C. aurantiacus* under the super strong promoter *P*_*cpc560*_ [16]. The growth of *Synechocystis* SM strain was almost identical as WT, and the 3-HP production observed in SM was approximately 691.58 ± 32.58 mg L^−1^ over 6 days’ cultivation, compared with no 3-HP production in WT (Fig. 1a, b), suggesting the production of 3-HP caused no visible metabolic burden or toxicity to cells. Through the 6 days’ cultivation, cells were collected for determination of differential metabolic responses of SM strain and WT using both iTRAQ-LC–MS/MS quantitative proteomic and LC–MS metabolomic analyses.

### Overview of quantitative proteomics and metabolomics

**Fig. 2:**
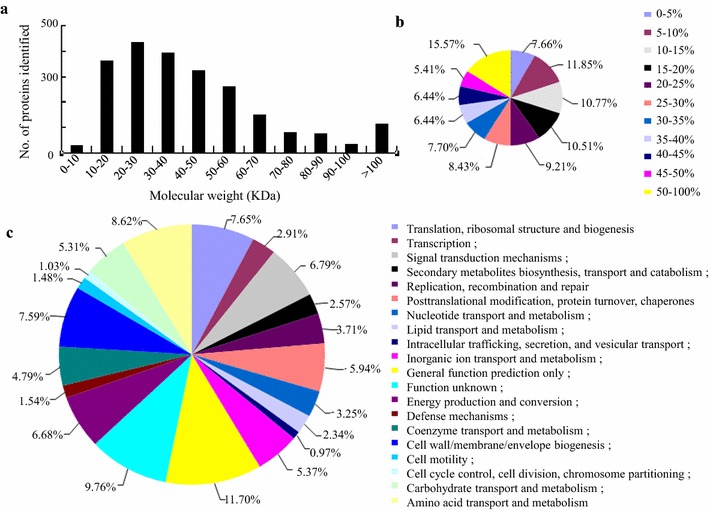
Distribution, coverage, and functional category of proteins identified in this study. **a** Distribution of protein identified among different molecular weights; **b** Coverage of proteins by the identified peptides; **c** Functional category coverage of the proteins identified

To investigate the metabolic responses of engineered SM strain to intracellular 3-HP production, cells of the WT and strain SM were harvested after 6 days’ cultivation at OD_730_ = 2.67 and 2.55, respectively (see Fig. 1a). A long cultivation time was used to maximize the metabolic responses of the *Synechocystis* cells to 3-HP. The proteomic analysis identified 374,706 spectra, among which 76,564 unique spectra met the strict confidence identification criteria and were matched to 2264 unique proteins. In addition, a good coverage was obtained for a wide MW range for proteins (Fig. 2a).

Most of the identified proteins were with good peptide coverage. ~81 % of the proteins were with more than 10 % of the sequence coverage and ~59 % were with more than 20 % of the sequence coverage (Fig. 2b). The proteins that were identified only in WT or engineered SM strain samples where ratio calculation is not available were eliminated from the analysis. Analysis of distribution among functional categories showed that “general function prediction only” was the top detected functional category, representing 11.7 % of all the identified protein (Fig. 2c). The high ratio of functionally unknown identified proteins is probably due to the fact that more than 33 % of proteins in the *Synechocystis* genome are hypothetical proteins [17]. Other frequently detected functional categories included “amino acid transport and metabolism” (8.62 %), “translation, ribosomal structure and biogenesis” (7.65 %), “cell wall/membrane/envelope biogenesis” (7.59 %), “energy production and conversion” (7.55 %) and “Signal transduction mechanisms” (6.79 %). Finally, proteomic analysis showed that 52 unique peptides belonged to malonyl-coenzyme A reductase was also identified in the SM strain, which confirmed its overexpression.

**Fig. 3:**
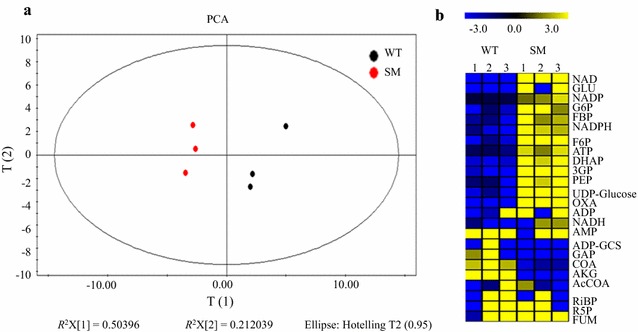
Targeted LC–MS metabolomic analysis. **a** PCA plots of the LC–MS metabolomic profiles of the WT and the 3-HP-producing SM strains; **b** Heatmap analysis of LC–MS metabolomic profiles of the WT and the 3-HP-producing SM strains

With the optimized LC–MS protocol established in our previous studies [18, 19], two sets of the metabolomic profiles each with 24 metabolites detected were obtained for WT and SM strains, respectively. The good separation of metabolomic profiles of the WT and the 3-HP-producing SM strain was also observed in the PCA plot, which suggested that metabolic changes occurred between WT and SM strains were significant (Fig. 3a).

### Metabolic responses to 3-HP synthesized internally in engineered *Synechocystis* strain

With a cutoff of 1.2-fold change and a *p* value of statistical significance less than 0.05, we found that 204 and 123 unique proteins were up- and down-regulated between the engineered SM and WT strains, respectively. We also determined that 15 and 2 central metabolites level were up- and down-regulated between the engineered SM and WT strains, respectively. The integrative proteomic and metabolomic analysis showed that several aspects of metabolism including energy metabolism, reducing power supply, central carbohydrate, nitrogen metabolism, protein synthesis, transporter, cofactors and amino acid metabolism and the stress response mechanism were enhanced in the SM strain after 3-HP production. Detailed description of responsive proteins and metabolites for each category was provided below.

#### Energy metabolism and reducing power supply

In cyanobacteria, the oxygenic photosynthesis involves two membrane protein complexes, photosystem I (PS I) and photosystem II (PS II) [20]. In the 3-HP engineered SM strain, three proteins Sll0629, Ssl0020 and Sll1051 that are related to photosynthesis were found up-regulated. Sll0629 is photosystem I subunit X which is an important component of photosystem I [21]. FedI (Ssl0020) is one of the most abundant ferredoxin products in cells [22]. Sll1051 is phycocyanobilin lyase CpcF, which is involved in phycocyanobilin attachment to the subunit of phycocyanin (CpcA) [23, 24]. In early studies of cellular responses to exogenous ethanol, hexane and butanol in *Synechocystis*, proteins involved in photosystem were also found up-regulated, which could be metabolic responses of cells to stress environments [10, 21, 25]. However, as no growth arrest was observed in the 3-HP-producing SM strain, it is speculative that enhanced photosynthesis and energy metabolism may be necessary for the high-level 3-HP production.

**Fig. 4:**
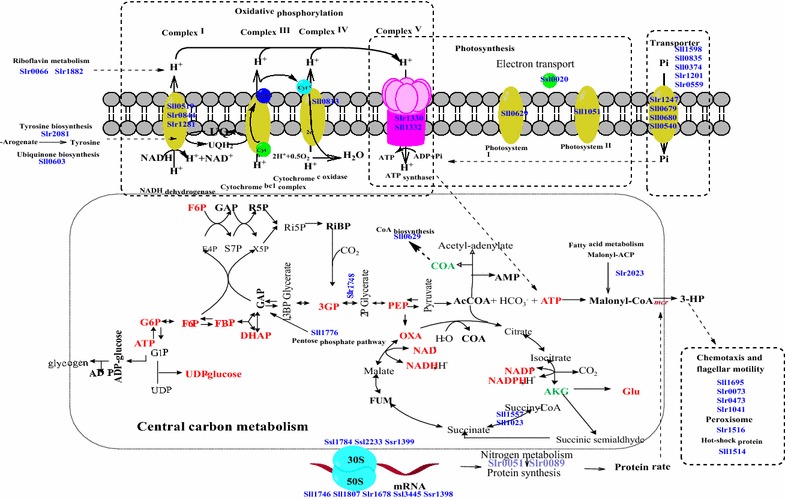
Schematic representation of metabolic responses to 3-HP synthesized internally in *Synechocystis*. Up-regulated proteins are indicated in the *figure*

The up-regulation of the abundance of a F0F1 ATP synthase subunit A (Sll1322) and a F0F1 ATP synthase subunit epsilon (Slr1330) was also observed in the 3-HP-producing strain SM [21]. The enhancement of ATP synthase, consistent with increased expression of proteins related to photosynthesis and oxidative phosphorylation, could increase the ATP supply to the engineered cells for the biosynthesis of 3-HP (Fig. 4). The enhanced photosynthesis and energy metabolism provided more energy to 3-HP production; meanwhile they also created increased needs for reducing power. Consistently, proteomic analysis showed that Sll0519, Slr1281, Slr0844 and Sll0813 related to oxidative phosphorylation were also up-regulated in strain SM. Sll0519 is annotated as NADH dehydrogenase subunit 1 NdhA [26], Slr1281 is annotated as NADH dehydrogenase I chain C [27], and Slr0844 is annotated as NAD(P)H-quinone oxidoreductase subunit F, respectively. They all belong to the NAD(P)H: quinone oxidoreductase (NDH-1) family that is a proton-translocating NAD(P)H: quinone oxidoreductase [28, 29], and functions to transfer electrons from an electron donor (usually NADH) to quinone to generate a proton motive force which is used for ATP synthesis [29]. In cyanobacteria, the NAD(P)H: quinone oxidoreductase (NDH-1) involves a variety of functions such as respiration, CO_2_ uptake and cyclic electron flow around PS I [30]. Sll0813 is cytochrome *c* oxidase subunit II that belongs to complex IV. Cytochrome *c* oxidase is a terminal respiratory oxidase which could accept electron and transmit it to oxygen [31].

#### Central carbohydrate metabolism

Using LC–MS-based metabolomic approach, 24 key metabolites were compared between the WT and the 3-HP-producing SM strains (Fig. 3b). Compared with WT, the analysis showed that the abundance of G6P, F6P, FBP, DHAP, 3GP and PEP were increased in SM strain. These metabolites involve in glycolysis/gluconeogenesis pathways. The end-product of glycolysis/gluconeogenesis pathway is also the initial substrate of tricarboxylic acid (TCA) cycle and 3-HP biosynthesis. F6P in glycolysis/gluconeogenesis pathway also participates in CO_2_ fixation. Thus, the enhancement of glycolysis/gluconeogenesis pathway may improve CO_2_ fixation, TCA and 3-HP production. The results were consistent with the proteomic analysis showing Slr1748, phosphoglycerate mutase that converts 3GP to 2GP [32], was up-regulated in SM strain. In addition, enhancement of glycolysis/gluconeogenesis pathway was also found in the quantitative proteomics analysis of ethanol producing strains of *Synechocystis*, in which phosphoglycerate kinase (Pgk), fructose-bisphosphate aldolase class1 (Fda), fructose-1,6-bisphosphatase class 1 (Slr0952) were up-regulated in response to ethanol production [25].

Deoxyribose-phosphate aldolase (Sll1776) in the pentose phosphate (PP) pathway was also found up-regulated. GAP could be synthesized through the catalysis of deoxyribose-phosphate aldolase. However, abundance of intracellular GAP was decreased in the 3-HP-producing strain as shown in Fig. 3. One of the possible explanations could be that GAP is necessary for both CO_2_ fixation and glycolysis/gluconeogenesis pathway, and the increased activity of these pathways led to increased or fast consumption of GAP in *Synechocystis*. Consistently, in ethanol-producing *Synechocystis* strain, glucose-6-phosphate 1-dehydrogenase (Zwf) functioned in pentose phosphate pathway was also up-regulated [25]. Response regulator of OmpR family Sll1330 was also up-regulated. As Sll1330 was found involved in the regulation of genes in glycolysis/gluconeogenesis pathway [33], its up-regulation could enhance the expression of the glycolysis/gluconeogenesis in the SM strain.

NADPH, NADP^+^, NADH, NADP^+^ and ATP were also increased in SM strain. They are involved in energy metabolism and reducing power supply in *Synechocystis*. The increased NADPH and ATP may also be caused by the enhancement of photosynthesis and oxidative phosphorylation. The proteomic analysis showed that two subunits of NAD(P) transhydrogenase Slr1239 and Slr1434 in strain SM were unchanged with a ratio of 1.033 and 1.069 fold compared with WT, respectively, suggesting that they are not affected by the 3-HP production in *Synechocystis*, which is consistent with our previous results that overexpression of *slr1239* and *slr1434* encoding genes in strain SM led to no significant improvement of 3-HP production [6].

The abundance of key precursor AcCOA was not changed in our metabolomic data (Fig. 3), which may be due to increased consumption of AcCOA into the biosynthesis of 3-HP and the TCA cycle. Consistent with this result, an early study has showed that in the PHB overproducing *Synechocystis* strain, intermediate metabolites for PHB such as acetyl-CoA was at low levels [14].

AKG is a key intermediate metabolite in TCA cycle, which is the hub of C-N metabolism in cells [34]. Metabolomic analysis showed a decreased abundance of AKG, which may be resulted from the increased amino acid synthesis in cells, such as Glu as revealed by metabolomic analysis (Fig. 4). In addition, the overexpression of key malonyl-CoA reductase (MCR) in strain SM could cost a large amount of amino acids. Proteomics analysis showed that ACP-S-malonyl transferase Slr2023 that converts malonyl-ACP to malonyl-CoA was up-regulated. Malonyl-CoA is the direct precursor for 3-HP production and the up-regulation of Slr2023 could result in increased supply of malonyl-CoA (Fig. 4).

#### Nitrogen metabolism and protein synthesis

In 3-HP-producing SM strain, Slr0051 and Slr0899 involved in nitrogen metabolism were up-regulated. Carbonic anhydrase Slr0051 could convert CO_2_ into HCO_3_^−^, which then could increase the HCO_3_^−^ concentration in *Synechocystis* [13], while HCO_3_^−^ is one precursor for malonyl-CoA production. Cyanate could be converted into CO_2_ and NH_3_ catalyzed by cyanate lyase Slr0899 in plants and cyanobacteria [35, 36]. Consistent with this result, carbonic anhydrase Slr0051 was also found up-regulated in ethanol producing *Synechocystis* [13]. Oxalate decarboxylase (Sll1358) involved in glyoxylate and dicarboxylate metabolism was also found up-regulated in the 3-HP-producing SM strain [37]. In addition, oxalate decarboxylase was also known for its role in pH homeostasis, so it is possible that Sll1358 may play a role in balancing pH in strain SM when 3-HP was produced. Eight proteins (*i.e.*, Sll1746, Sll1807, Slr1678, Ssl3445, Ssr1398, Ssl1784, Ssl2233 and Ssr1399) involved in ribosome synthesis were also up-regulated. Sll1746, Sll1807, Slr1678, Ssl3445 and Ssr1398 were annotated as 50S ribosomal protein subunits and Ssl1784, Ssl2233 and Ssr1399 were annotated as 30S ribosomal protein subunits. The results were consistent with the previous transcriptomic analysis of cellular response to ethanol production in *Synechocystis*, in which the *rps8* (*sll1809* encoding ribosomal protein S8) mRNA level was found increased by 3 to sixfold during the whole ethanol production process [12].

#### Transporter

In 3-HP-producing SM strain, 11 transporters were found up-regulated, including Slr1247, Sll0540, Sll0679, Sll0680, Sll1598, Sll0385, Sll1699, Sll0374, Sll1270, Slr0559 and Slr1201. Phosphorus is necessary to the ATP and NADPH synthesis, and in *Synechocystis* transporters with three associated Pi-binding proteins (PBPs) have been identified, they are PstS1 (Sll0680), PstS2 (Slr1247) and SphX (Sll0679) [38]. Recently, the fourth potential PBP was also proposed as Sll0540 based on BLAST analysis [38]. SphX encoded by *sll0679* is a phosphate transport system substrate-binding protein. In *Synechocystis*, the expression level of *sll0679* was increased about 37-fold under the Pi stress conditions. Under nutritional deficiency condition, the expression of *sll0679* gene was increased, which improved the uptake of phosphorus [38]. The up-regulation of all four PBPs in SM strain was probably due to the increased consumption of Pi in the form of NAPDH or ATP.

Up-regulated Sll1598 is annotated as a manganese transport system substrate-binding protein. The expression of the *mntCAB* operon (*sll1598*-*1600*) was increased by a low concentration of Mn^2+^ [39]. In *Synechocystis*, four manganese ions were associated with the PS II active center [40], suggesting key role of manganese in photosynthesis. It is speculative that the up-regulation of Sll1598 could enhance the photosynthesis for better supply of ATP and NADPH into the 3-HP production.

Up-regulated Sll0385 is annotated as cobalt transport system ATP-binding protein. Cobalt is part of coenzyme B_12_ cofactor that involves both methyl group rearrangements and transfer reactions [41]. Although some cyanobacteria could synthesize vitamin B_12_-related compounds by themselves, study has found that B_12_ is required for growth in *Synechococcus* sp. PCC 7002 [42]. In *Synechocystis*, it has been previously found that the expression of Sll0385 was increased by the cold stress condition [43]. In *Synechocystis* vitamin B_12_ is relevant to the synthesis of amino acids especially methionine. *Synechocystis* can synthesize B_12_ de novo and utilize it as cofactor for cobalamin-dependent methionine synthase [43]. In this case, it is speculative that the overexpression of gene *sll0385* may enhance amino acids synthesis that provides more materials to the synthesis of malonyl-CoA reductase.

Up-regulated Sll1699 is annotated as a peptide transport system substrate-binding protein. In addition to primary role in cell nutrition, peptide transport systems are also involved in various signaling processes in microbes [44]. The peptide transporters could also help the bacteria sense the local environment and adapt to these conditions through adjusting expression of specific genes [45]. Role of the up-regulated Sll1699 caused by 3-HP production is still not immediately clear and may worth further investigation.

Finally, urea transport system ATP-binding protein Sll0374, urea transport system permease protein Slr1201, arginine/lysine/histidine/glutamine transport system substrate-binding and permease protein Sll1270 and neutral amino acid transport system substrate-binding protein Slr0559 were also up-regulated, although their roles in metabolic responses to 3-HP synthesis is yet to be established.

#### Cofactors and amino acid metabolism

Slr0066 and Slr1882 belonged to riboflavin metabolism were up-regulated in SM strain. Slr0066 is annotated as an riboflavin biosynthesis protein [46] and Slr1882 is annotated as an riboflavin kinase [47]. The active forms of riboflavin, such as flavin mononucleotide (FMN) and flavin adenine dinucleotide (FAD), function as cofactors for a variety of oxidative phosphorylation reactions. Cysteine desulfurase Slr0387 involved in thiamine metabolism was also up-regulated [48]. As a cofactor of pyruvate carboxylase and other carboxylases, thiamine involves in pentose phosphate pathway that itself was also up-regulated in 3-HP-producing cells. Slr1598 belonged to lipoic acid metabolism was also up-regulated in strain SM. Slr1598 is annotated as lipoic acid synthetase [49]. Lipoic acid is cofactor for the pyruvate dehydrogenase complex and α-ketoglutarate dehydrogenase complex. These complexes are essential in the citric acid cycle. Slr0078 and Slr1979 related to folate biosynthesis was up-regulated in strain SM. Slr0078 is annotated as 6-carboxytetrahydropterin synthase and Slr1979 is annotated as anthranilate synthase component I. Folate is necessary for DNA synthesis, RNA synthesis and amino acid production which was necessary to *Synechocystis* normal growth and 3-HP production [50].

Arogenate dehydrogenase Slr2081 involved in tyrosine biosynthesis pathway was up-regulated. The enzyme catalyzes the conversion of arogenate to tyrosine. In addition, 2-succinyl-5-enolpyruvyl-6-hydroxy-3-cyclohexene-1-carboxylate synthase Sll0603 was also found up-regulated, the enzyme is a key enzyme in the biosynthetic pathway of PS I related phylloquinone [51]. Proteomics analysis showed that carboxynorspermidine decarboxylase Sll0873 involved in arginine and proline metabolism could be converted into spermidine which is necessary in the biosynthesis of β-alanine. Up-regulated urease subunit gamma Slr1256 was involved in arginine biosynthesis. The results were consistent with a study on cellular response of *E. coli* to 3-HP, where proteins involved in amino acid biosynthesis were differentially regulated during adaptation to 3HP [52].

#### Common stress response

Chemotaxis and flagellar motility are essential mechanisms and through the mechanisms bacteria could adapt to and survive in stress environments [21, 53]. Our proteomic analysis found that several proteins involved in motility function were up-regulated in *Synechocystis*, including a type IV pilus assembly protein Sll1695, a type IV pilus sensor histidine kinase Slr0073 [54], were up-regulated. Type IV pilus is necessary to cell motility in *Synechocystis*. A two-component system response regulator Slr1041 (PilG) that is also involved in pilus motility [54], a sensor kinase Slr0473 of chemotaxis family, and a periplasmic WD repeat-containing protein Sll1491 that was found involved in spore maturation and cell motility of *Myxococcus xanthus* [55].

Hot shock protein Sll1514 involved in refolding of lipoproteins (RlpA, Slr0423; RepA, Ssl3177) was also up-regulated. The protein was previously found related to cell wall stability in *Synechocystis* [32]. As a common stress response strategy, early studies have showed that heat-shock proteins were responsive to tolerance to butanol in both native and non-native producing microorganisms [56, 57]. Several cyanobacterial heat-shock proteins were also found previously responsive to various stress conditions, such as *htpG* related to thermo tolerance [58], an amphitropic small heat-shock proteins (sHsps) to elevated resistance against UV-B damage [59], and expression of heat-shock genes *groES*, *groEL1* and *groEL2* low-temperature-inducible in *Synechocystis* [60]. In a proteomic analysis of engineered PHB-producing *E. coli*, three heat shock proteins, GroEL, GroES, and DnaK, were also significantly up-regulated [61]. Early studies have found that cells stressed by solvents (i.e., phenol, ethanol) generated highly reactive oxygen species (ROS) toxic to cells [62]. The oxidative stress response was also observed in *E. coli* treated with *n*-butanol, where the *cyo*, *nuo*, and *sdh* operons, *sodA* encoding a superoxide dismutase, and *yqhD* encoding an alcohol dehydrogenase were up-regulated [21, 63]. Consistently, our analysis showed that superoxide dismutase Slr1516 was up-regulated in SM strain.

Our proteomics analysis showed that d-alanyl-d-alanine carboxypeptidase Slr1924 was up-regulated. Slr1924 was involved in peptidoglycan biosynthesis. Peptidoglycan is a key component in cell wall. So, this up-regulated protein may be involved in protection of cells under stress environments.

Together, the analysis showed that when 3-HP was synthesized at a high level, abundances of metabolites in glycolysis/gluconeogenesis pathway were increased, and proteins in glycolysis/gluconeogenesis pathway, pentose phosphate pathway and fatty acid biosynthesis pathway were up-regulated in order to supply more precursor malonyl-CoA and acetyl-CoA. In addition, as NADPH and ATP were necessary to the 3-HP production and cell normal growth, metabolism involved in photosynthesis, oxidative phosphorylation and abundance of metabolites in TCA cycle were increased. Moreover, metabolisms involved in tyrosine, riboflavin, ubiquinone metabolic pathways and transporters were also up-regulated, consistent with a previous quantitative proteomics analysis of cellular responses to ethanol synthesized internally in *Synechocystis* [12, 13].

Although first conducted for 3-HP-producing cyanobacteria, omics analysis of non-photosynthetic systems carrying the 3-HP biosynthetic pathway has been performed before. For example, to improve *E. coli* resistance to 3HP and reduce the total production cost in industrial applications, a two-dimensional gel (2D-Gel) electrophoresis based proteomic analysis has been applied to determine variations in protein expression levels exposed to sub-lethal concentration of 3-HP [52]. The results showed that 46 proteins were up-regulated, while 23 proteins were repressed. The up-regulated proteins were classified into several categories based on their functions, and the top three largest categories are amino acids metabolism, energy metabolism, and ATP biosynthesis [52], which are consistent with our results presented above. The MCR coding gene from *C. aurantiacus* was also integrated into the genome of *Saccharomyces cerevisiae*, and a 3-HP production of 9.8 ± 0.4 g L^−1^ after 100 h was reported [64]. RNA-seq based transcriptome analysis of the non-producing and the best 3-HP-producing yeast strains was performed. The results showed that genes involved in the PP pathway and tricarboxylic acid (TCA) cycle were up-regulated which may lead to improved NADPH availability in the cytosol for 3-HP production. In addition, genes related to redox metabolism were differentially regulated, probably due to the changing NADPH demands for 3HP biosynthesis. Moreover, significant changes in transcription of genes related to glycolytic pathway, amino acid synthesis and transport were also observed in the 3-HP-producing yeast [64]. Finally, a proteome analysis of metabolically engineered PHB-producing *E. coli* showed that the increased cellular demand of acetyl-CoA and NADPH for PHB biosynthesis resulted in the increased synthesis of two enzymes of the glycolytic pathway and one enzyme of the Entner-Doudoroff pathway [61]. These previous studies were mostly consistent with our analysis with the engineered 3-HP-producing *Synechocystis*, such as increased abundance of proteins and metabolites involved in PP pathway, tricarboxylic acid (TCA) cycle, glycolytic pathway, oxygenic photosynthesis and oxidative phosphorylation.

### RT-PCR confirmation of abundance change of responsive proteins

To confirm abundance changes of responsive proteins revealed by iTRAQ quantitative proteomic analysis, we selected 20 genes based on their expression levels and their regulatory patterns in SM strain (i.e., up-, or down-regulation) for a quantitative RT-PCR analysis. Among them, 10 proteins were down-regulated (i.e., Sll0992, Ssl2667, Ssr2061, Slr0447, Sll0541, Slr1019, Sll0482, Slr1856, Sll1087 and Slr1200) and 10 proteins were up-regulated (i.e., Slr0420, Sll1773, Slr0431, Slr1227, Sll1869, Sll0385, Sll1699, Slr0844, Sll1598 and Sll1491) according to the iTRAQ proteomic analysis, respectively. Comparative RT-PCR analysis between the 3-HP-producing SM strain and WT showed overall good consistence between RT-qPCR and proteomic data (Table 1), and correlation coefficient between RT-qPCR and proteomics data was 0.75, suggesting a good quality of this proteomic data.

**Table 1:** Comparison of ratios calculated from iTRAQ proteomics and RT-PCR analyses

	Gene ID	Proteomics ratio	RT-PCR ratio	Function description
Up-regulated	*slr0420*	2.76 ± 0.05	4.05 ± 1.88	Hypothetical protein
	*sll1773*	2.51 ± 0.05	4.24 ± 1.65	Conserved hypothetical protein
	*slr0431*	2.42 ± 0.09	2.61 ± 0.83	Conserved hypothetical protein
	*slr1227*	2.02 ± 0.36	0.85 ± 0.15	OMP1 precursor
	*sll1869*	1.71 ± 0.16	2.71 ± 0.46	A-subunit oxygenase
	*sll0385*	1.35 ± 0.02	0.90 ± 0.46	ABC transporter
	*sll1699*	1.34 ± 0.04	2.22 ± 0.34	Extracellular solute binding
	*slr0844*	1.37 ± 0.06	0.88 ± 0.25	NADH-plastoquinone oxidoreductase chain 5
	*sll1598*	1.24 ± 0.02	1.73 ± 0.57	Iron (chelated) ABC transporter,
	*sll1491*	1.32 ± 0.49	4.35 ± 0.03	Periplasmic WD repeat-containing protein
Down-regulated	*sll0992*	0.42 ± 0.01	0.80 ± 0.08	Conserved hypothetical protein
	*ssl2667*	0.51 ± 0.03	0.68 ± 0.05	Putative NifU protein
	*ssr2061*	0.44 ± 0.03	0.65 ± 0.09	Glutaredoxin 3 (*grx3*)
	*slr0447*	0.49 ± 0.02	0.68 ± 0.23	Urea/short-chain amide ABC transporter
	*sll0541*	0.65 ± 0.13	0.75 ± 0.29	*Delta*-9 desaturase
	*slr1019*	0.56 ± 0.08	1.75 ± 0.35	PhzF
	*sll0482*	0.75 ± 0.21	2.06 ± 0.98	Lipase precursor
	*slr1856*	0.79 ± 0.11	2.21 ± 0.57	Sigma regulatory factor
	*sll1087*	0.83 ± 0.10	0.89 ± 0.32	Sodium-coupled permease
	*slr1200*	0.75 ± 0.05	1.08 ± 0.20	Urea/short-chain amide ABC transporter, permease protein

### Overexpression and validation of genes relevant to 3-HP production

To validate the responses uncovered by the proteomic and metabolomic analysis and their relevance to 3-HP production, attempt was made to utilize the information for further modifying 3-HP-producing SM strain. Towards this goal, encoding genes of 11 responsive proteins were selected as preliminary targets for overexpression in SM strain. These genes were selected first based on the significance of their up- or down-regulation in the proteomics data, among which Sll0385, Sll1598, Sll0679, Slr0473, Sll1869, Sll1699, Slr0844, Slr1227, Slr1805 and Sll1491 were up-regulated, while Sll1087 was down-regulated at a protein level in SM strain (Table 2). In addition, the regulation patterns of the selected proteins were also confirmed by RT-qPCR analysis (data not shown). Furthermore, the proteins were selected for validation also based on a possible functional relevance to 3-HP production with a focus on various putative transporters, as recent studies showed that transporters specifically have emerged as a powerful category of proteins that bestow tolerance and often improve production in engineered microbes [65, 66].

**Table 2:** 3-HP production in engineered *Synechocystis* strains

Strains	Description	Proteomics ratio	3-HP production (mg L^−1^)
SM (control)	*P*_*cpc560*_ and gene *mcr* integrated into *Synechocystis*		687.80 ± 19.12
SM-*sll1869*	CbaB; 3-chlorobenzoate-3,4-dioxygenase	1.71 ± 0.16	657.22 ± 30.37
SM-*sll0385*	ABC transporter	1.35 ± 0.02	718.64 ± 23.35
SM-*sll1699*	ABC transporter substrate-binding protein	1.34 ± 0.04	699.31 ± 32.99
SM-*slr0844*	NdhF; NAD(P)H-quinone oxidoreductase subunit F	1.37 ± 0.06	646.85 ± 23.09
SM-*sll1598*	Iron (chelated) ABC transporter	1.24 ± 0.02	732.18 ± 25.21
SM-*sll1491*	Periplasmic WD repeat-containing protein	1.24 ± 0.02	633.86 ± 24.09
SM-*sll1087*	Sodium-coupled permease	0.83 ± 0.10	651.13 ± 28.37
SM-*sll0679*	Phosphate-binding periplasmic protein	1.21 ± 0.06	752.22 ± 29.36
SM-*slr1227*	Outer membrane protein insertion porin family	2.02 ± 0.36	689.17 ± 35.83
SM-*slr1805*	Sensory transduction histidine kinase	1.37 ± 0.05	694.59 ± 27.55
SM-*slr0473*	Phytochrome	1.21 ± 0.01	669.39 ± 23.66

For the 11 selected genes, 5 were related to transporting function of cells. Gene *sll0385* encodes a cobalt/nickel transport system ATP-binding protein relevant to the cobalamin synthesis [67], gene *sll1598* encodes a manganese transport system substrate-binding protein [36], gene *sll0679* encodes a phosphate transport system substrate-binding protein [38], gene *slr0844* encodes a NAD(P)H-quinone oxidoreductase subunit F [29], gene *sll1087* encoding a sodium-coupled permease belonged to solute: Na^+^ symporter in *Synechocystis*, respectively.

For the remaining 6 genes, gene *slr0473* encodes a sensor histidine kinase of chemotaxis family [68], gene *sll1869* encodes 3-chlorobenzoate-3,4-dioxygenase [69], *sll1699* encodes a peptide transport system substrate-binding protein that plays an important role in various signaling processes and the defense against cationic antimicrobial peptides [44], gene *slr1227* encodes an outer membrane protein insertion porin family that may also be involved in peptides transport [70], gene *slr1805* encodes a histidine kinase related to perceiving osmotic pressure and transmitting the signal into the cells [71], and gene *sll1491* encodes a periplasmic WD repeat-containing protein which played a role in stress environment adaption [21, 55], respectively.

**Fig. 5:**
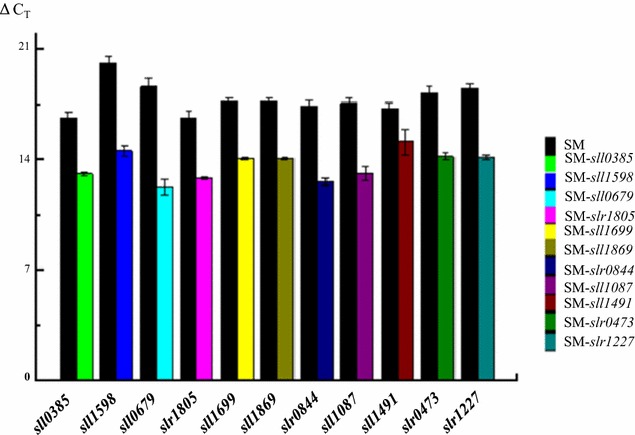
RT-qPCR analysis of the expression level of selected overexpressed genes in SM strain

Coding sequences of the 11 selected genes were amplified from the *Synechocystis* chromosomal DNA and overexpressed using a vector pXT37b in the *Synechocystis* SM strain. Before phenotypic and 3-HP biosynthesis were analyzed, detection of 3-HP production, RT-qPCR analysis of the expression levels of 11 targeted gene was conducted and the results confirmed that the expression level was all increased for the 11 genes by 4- to 82-fold (Fig. 5). Although no growth difference was observed for the WT and the 11 engineered *Synechocystis* strains (Additional file 1: Figure S1), analysis of 3-HP production showed that 3-HP production in strain SM-*sll0385*, SM-*sll1598* and SM-*sll0679* was increased to 735.14 ± 18.13, 715.36 ± 18.76 and 738.90 ± 29.94 mg L^−1^, respectively, representing increases of 3–6 % when compared with the original strain SM (Table 2). As transporting and availability of these metals are closely related to photosynthesis, oxidative phosphorylation, amino acids synthesis, it is speculative that these metabolisms were important for the further production improvement of 3-HP in *Synechocystis*. In addition, the increase of 3-HP synthesis after overexpressing single gene was not significant, suggesting that the complicated metabolic re-wiring involved in multiple genes may be necessary for enhancing 3-HP production. Furthermore, for the remaining 8 gene targets we evaluated, no significant increase of 3-HP production was observed. Although the reasons yet to be determined, it is possible that either they are responsive as secondary metabolic responses to 3HP internally synthesized, or a functional coordination of these genes with other genes is necessary in *Synechocystis* so that overexpression of only a single gene resulted no visible effect on 3-HP production.

## Conclusions

In this study, the metabolic responses of *Synechocystis* to 3-HP internally produced were first determined using a quantitative proteomics approach with iTRAQ-LC–MS/MS and LC–MS technologies. The analyses showed that proteins related to oxidative phosphorylation, photosynthesis, ribosome, central carbon metabolism, two-component system, and ABC-type transporters were differentially regulated. To confirm the information obtained from the proteomics and metabolomics analysis, we constructed gene expression strains of selected responsive genes and determined the effects on 3-HP production, and the results showed that the overexpression of three transporter genes putatively involved in cobalt/nickel, manganese and phosphate transporting (i.e., *sll0385*, *sll1598* and *sll0679*) could lead to increased production of 3-HP in *Synechocystis*. The study not only presented a list of tentative gene targets for further engineering the 3-HP-producing strains, but also demonstrated that integrating systems biology analysis of chemical-producing cells could be an effective way to identify metabolic responses to the production and could lead to rational design and engineering of the high efficiency strains.

## Methods

### 3-HP production growth conditions

3-HP *Synechocystis* producing SM strain and its derivatives were grown in 100-mL flasks which contained 20-mL BG11 medium under the normal growth condition [72] until they reached an OD_730_ value of 1.0. Then the cells were collected by centrifugation (900×*g*) and washed once with BG11 media, before being re-suspended in the fresh BG11 media (10 mL) to a cell density of OD_730_ approximately 1.5. The cells were incubated in a shaking bed (150 rpm) at 30 °C with light intensity of 50 μmol photons m^−2^ s^−1^. 0.5 mL 1.0 M NaHCO_3_ was added to each flask every 24 h, and the culture medium was adjusted to pH 7.5 using 10 N HCl [71]. The cells were grown for up to 6 days. Cell growth (OD_730_) and the 3-HP biosynthesis were measured through the growth time course. Three biological replicates were independently established for each experiment, and three analytical replicates were conducted for each sample [6].

### Construction and analysis of overexpression strains

All strains used and constructed in this study are listed in Additional file 2: Table S3. All primers used are provided in Additional file 2: Table S4. Plasmid pXT37b for gene expressing was kindly provided by Prof. Xuefeng Lu of Qingdao Institute of Bioenergy and Bioprocess Technology of Chinese Academy of Sciences [73]. In this study, the promoter of plastocyanin (*P*_*petE*_) was first replaced by phycocyanin beta chain (*P*_*cpcB*_) promoter. The ORF of *sll1869*, *sll0385*, *sll1699*, *slr0844*, *sll1598*, *sll1491*, *sll1087*, *sll0679*, *slr1227*, *slr1805* and *slr0473* were PCR amplified and sub-cloned into *Nde*I/*Xho*I site of the modified pXT37b, resulting in pTX-*sll1869*, pTX-*sll0385*, pTX-*sll1699*, pTX-*slr0844*, pTX-*sll1598*, pTX-*sll1491*, pTX-*sll1087*, pTX-*sll0679*, pTX-*slr1227*, pTX-*slr1805* and pTX-*slr0473*, respectively. Plasmid pTX-*sll1869*, pTX-*sll0385*, pTX-*sll1699*, pTX-*slr0844*, pTX-*sll1598*, pTX-*sll1491*, pTX-*sll1087*, pTX-*sll0679*, pTX-*slr1227*, pTX-*slr1805* and pTX-*slr0473* were introduced into SM strain by natural transformation and strain SM-*sll1869*, SM-*sll0385*, SM-*sll1699*, SM-*slr0844*, SM-*sll1598*, SM-*sll1491*, SM-*sll1087*, SM-*sll0679*, SM-*slr1227*, SM-*slr1805* and SM-*slr0473* were obtained, respectively. To achieve complete chromosome segregation, engineering strains were passed several times on fresh BG11 plates supplemented with 10 µg/mL spectinomycin. Homologous integration of the expressing cassette and complete segregation were confirmed by PCR analysis [73].

### Protein preparation

Protein preparation was performed as described previously [10, 21, 25]. Briefly, the cells were suspended in the lysis buffer and sonicated in ice. The proteins were reduced with 10-mM DTT then alkylated by 55-mM iodoacetamide. Protein mixtures were precipitated at −20 °C overnight. After dissolution in 0.5 M TEAB (Applied Biosystems, Milan, Italy) the pellet was sonicated in ice. Then mixtures were centrifuged at 30,000×*g* 4 °C and an aliquot of the supernatant was taken for determination of protein concentration by coomassie blue staining [74] and normalized. The proteins in the supernatant were kept at −80 °C for further analysis.

### iTRAQ labeling

Total protein (100 μg) taken out of each sample solution was digested with Trypsin Gold (Promega, Madison, WI, USA). The peptides were then dried by vacuum centrifugation and reconstituted in 0.5 M TEAB. Samples were labeled with the iTRAQ tags according to the manufacture’s protocol as described in our previous study [10, 21, 25]. Strong cation exchange chromatography (SCX) was performed with a LC-20AB HPLC Pump system (Shimadzu, Kyoto, Japan). The iTRAQ-labeled peptide mixtures were reconstituted and loaded onto a 4.6 × 250 mm Ultremex SCX column that contained 5-μm particles (Phenomenex, Torrance, US). The peptides were eluted. And the eluted peptides were pooled into 20 fractions, desalted with a Strata X C18 column (Phenomenex, Torrance, US) and vacuum-dried.

### LC–ESI–MS/MS analysis

Each fraction was re-suspended and centrifuged; the final peptide concentration was about 0.5 μg/μL on average. 10-μL supernatant was loaded on a LC-20AD nanoHPLC (Shimadzu, Kyoto, Japan) by the autosampler onto a 2-cm C18 trap column. Then, the peptides were eluted onto a 10-cm analytical C18 column (inner diameter 75 μm) packed in-house. Data acquisition was performed as described previously [10, 21, 25] with a TripleTOF 5600 System (AB SCIEX, Concord, ON) fitted with a Nanospray IIIsource (AB SCIEX, Concord, ON) and a pulled quartz tip as the emitter (New Objectives, Woburn, MA). The MS was operated with a RP greater than or equal to 30,000 FWHM for TOF MS scans. For IDA, survey scans were acquired in 250 ms and as many as 30 product ion scans were collected if exceeding a threshold of 120 counts per second (counts/s) and with a 2+ to 5+ charge-state. A sweeping collision energy coupled with iTRAQ adjust rolling collision energy was applied to all precursor ions for collision-induced dissociation. Dynamic exclusion was set for 1/2 of peak width (15 s), and then the precursor was refreshed off the exclusion list [10, 21, 25].

### Data analysis

Original data obtained from the Orbitrap was converted into MGF files with Proteome Discoverer 1.2 (PD 1.2, Thermo Fisher Scientific), and the MGF file was searched. Proteins identification was performed using Mascot search engine (Matrix Science, London, UK; version 2.3.02) with a *Synechocystis* sequence database. Protein identification was conducted according to our previous publication [10, 21, 25]. For protein quantification, a protein contains at least two unique spectra. The confident ratio change with *p* values <0.05, and only fold changes of >1.2 was considered as significant [10, 21, 25]. The mass spectrometry proteomics data have been deposited to the ProteomeXchange Consortium via the PRIDE [75] partner repository with the dataset identifier PXD004974.

### Function method description

Proteins functional annotation was conducted using Blast2GO program against the non-redundant protein database (NR; NCBI). The KEGG database (http://www.genome.jp/kegg/) and the COG database (http://www.ncbi.nlm.nih.gov/COG/) were used to classify and group this identified proteins [76].

### LC–MS-based metabolomics analysis

The LC–MS-based targeted metabolomic analysis was performed according to the protocol described previously [18, 19]. A total of 24 metabolites were selected in this study [77]. Primarily all metabolomic profile data were normalized through the internal control compound and the cell numbers of the samples. Then data was subjected to partial least squares discriminant analysis (PLS-DA) which is a supervised clustering or classification method using software SIMCA-P 11.5 [78]. Each condition analysis consisted of three biological replicates and two technical replicates.

### Quantitative real-time RT-PCR analysis

Twenty proteins significantly up- or down-regulated were chosen for RT-qPCR validation. Approximately 1.67 × 10^8^ *Synechocystis* cells (assuming OD_730_ of 0.6 equals to 10^8^ cells/mL were collected by centrifugation at 17,000×*g*, 4 °C for 1 min [79]. RNA extraction and RT-qPCR analysis were conducted according to the method described previously [80]. The relative abundance of different mRNA molecules could be estimated using 2^−∆∆CT^; the higher the ^∆^C_T_ value is, the less abundant the corresponding mRNA, as described in previous study [81].

## Additional files

10.1186/s13068-016-0627-6 Comparison of cell growth of the WT and the engineered *Synechocystis* strains in this study.

10.1186/s13068-016-0627-6 Proteins up-regulated in 3-HP production SM strain. **Table S2.** Hypothetical and unclassified proteins up-regulated in 3-HP production SM strain. **Table S3**. Strains and plasmids used in this study. **Table S4**. Primers used in this study.

## Abbreviations

AcCOA: acetyl coenzyme A
ADP: adenosine 5′-diphosphate
ADP-GCS: adenosine-5′-diphosphoglucose
AKG: α-ketoglutaric acid
ATP: adenosine 5′-triphosphate (ATP)
AMP: adenosine 5′-monophosphate
COA: coenzyme A hydrate
DHAP: dihydroxyacetone phosphate
FBP: d-fructose 1, 6-bisphosphate
FUM: sodium fumarate dibasic
F6P: d-fructose 6-phosphate
GAP: DL-glyceraldehyde 3-phosphate
GLU: l-glutamic acid
G6P: d-glucose 6-phosphate
iTRAQ: isobaric tag for relative and absolute quantitation
KEGG: Kyoto Encyclopedia of Genes and Genomes
LC–MS/MS: liquid chromatography-tandem mass spectrometry
NAD: α-nicotinamide adenine dinucleotide
NADH: NADH
NADP: NADP
NADPH: NADPH
OXA: oxaloacetic acid
PEP: phospho (enol) pyruvic acid
RiBP: d-ribulose 1, 5-bisphosphate
R5P: d-ribose 5-phosphate
UDP-GCS: uridine 5′-diphosphoglucose
3PG: d-3-phosphoglyceric acid

## References

[1] Kim K, Kim S-K, Park Y-C, Seo J-H (2014). Enhanced production of 3-hydroxypropionic acid from glycerol by modulation of glycerol metabolism in recombinant *Escherichia coli*. Bioresour Technol.

[2] Bozell JJ, Petersen GR (2010). Technology development for the production of biobased products from biorefinery carbohydrates—the US Department of Energy’s “top 10” revisited. Green Chem.

[3] Kumar V, Ashok S, Park S (2013). Recent advances in biological production of 3-hydroxypropionic acid. Biotechnol Adv.

[4] Raj SM, Rathnasingh C, Jo JE, Park S (2008). Production of 3-hydroxypropionic acid from glycerol by a novel recombinant *Escherichia coli* BL21 strain. Process Biochem.

[5] Rathnasingh C, Raj SM, Lee Y, Catherine C, Ashok S, Park S (2012). Production of 3-hydroxypropionic acid via malonyl-CoA pathway using recombinant *Escherichia coli* strains. J Biotechnol.

[6] Wang Y, Sun T, Gao X, Shi M, Wu L, Chen L (2015). Biosynthesis of platform chemical 3-hydroxypropionic acid (3-HP) directly from CO_2_ in cyanobacterium *Synechocystis* sp. PCC 6803. Metab Eng.

[7] Vu TT, Hill EA, Kucek LA, Konopka AE, Beliaev AS, Reed JL (2013). Computational evaluation of *Synechococcus* sp. PCC 7002 metabolism for chemical production. J Biotechnol.

[8] Feist AM, Zielinski DC, Orth JD, Schellenberger J, Herrgard MJ, Palsson BØ (2010). Model-driven evaluation of the production potential for growth-coupled products of *Escherichia coli*. Metab Eng.

[9] Nogales J, Gudmundsson S, Knight EM, Palsson BO, Thiele I (2012). Detailing the optimality of photosynthesis in cyanobacteria through systems biology analysis. Proc Natl Acad Sci USA.

[10] Qiao J, Wang J, Chen L, Tian X, Huang S, Ren X (2012). Quantitative iTRAQ LC–MS/MS proteomics reveals metabolic responses to biofuel ethanol in cyanobacterial *Synechocystis* sp. PCC 6803. J Proteome Res.

[11] Wang J, Chen L, Huang S, Liu J, Ren X, Tian X (2012). RNA-seq based identification and mutant validation of gene targets related to ethanol resistance in cyanobacterial *Synechocystis* sp. PCC 6803. Biotechnol Biofuels.

[12] Dienst D, Georg J, Abts T, Jakorew L, Kuchmina E, Börner T (2014). Transcriptomic response to prolonged ethanol production in the cyanobacterium *Synechocystis* sp. PCC 6803. Biotechnol Biofuels.

[13] Borirak O, Koning LJ, Woude AD, Hoefsloot HC, Dekker HL, Roseboom W (2015). Quantitative proteomics analysis of an ethanol-and a lactate-producing mutant strain of *Synechocystis* sp. PCC6803. Biotechnol Biofuels.

[14] Hondo S, Takahashi M, Osanai T, Matsuda M, Hasunuma T, Tazuke A (2015). Genetic engineering and metabolite profiling for overproduction of polyhydroxybutyrate in cyanobacteria. J Biosci Bioeng.

[15] Gudmundsson S, Nogales J (2015). Cyanobacteria as photosynthetic biocatalysts: a systems biology perspective. Mol BioSyst.

[16] Zhou J, Zhang H, Meng H, Zhu Y, Bao G, Zhang Y (2014). Discovery of a super-strong promoter enables efficient production of heterologous proteins in cyanobacteria. Sci Rep.

[17] Gao L, Pei G, Chen L, Zhang W (2015). A global network-based protocol for functional inference of hypothetical proteins in *Synechocystis* sp. PCC 6803. J Microbiol Method.

[18] Bennette NB, Eng JF, Dismukes GC (2011). An LC–MS-based chemical and analytical method for targeted metabolite quantification in the model cyanobacterium *Synechococcus* sp. PCC 7002. Anal Chem.

[19] Su Y, Wang J, Shi M, Niu X, Yu X, Gao L (2014). Metabolomic and network analysis of astaxanthin-producing *Haematococcus pluvialis* under various stress conditions. Bioresour Technol.

[20] Zhang P, Frankel LK, Bricker TM (2013). Integration of apo-α-phycocyanin into phycobilisomes and its association with FNR L in the absence of the phycocyanin α-subunit lyase (CpcF) in *Synechocystis* sp. PCC 6803. PLoS ONE.

[21] Tian X, Chen L, Wang J, Qiao J, Zhang W (2013). Quantitative proteomics reveals dynamic responses of *Synechocystis* sp. PCC 6803 to next-generation biofuel butanol. J Proteom.

[22] Poncelet M, Cassier-Chauvat C, Leschelle X, Bottin H, Chauvat F (1998). Targeted deletion and mutational analysis of the essential (2Fe–2S) plant-like ferredoxin in Synechocystis PCC 6803 by plasmid shuffling. Mol Microbiol.

[23] Fairchild CD, Zhao J, Zhou J, Colson SE, Bryant DA, Glazer AN (1992). Phycocyanin alpha-subunit phycocyanobilin lyase. Proc Natl Acad Sci USA.

[24] Zhou J, Gasparich GE, Stirewalt VL, De Lorimier R, Bryant D (1992). The cpcE and cpcF genes of *Synechococcus* sp. PCC 7002. Construction and phenotypic characterization of interposon mutants. J Biol Chem.

[25] Liu J, Chen L, Wang J, Qiao J, Zhang W (2012). Proteomic analysis reveals resistance mechanism against biofuel hexane in *Synechocystis* sp. PCC 6803. Biotechnol Biofuels.

[26] Pisareva T, Kwon J, Oh J, Kim S, Ge C, Wieslander Å (2011). Model for membrane organization and protein sorting in the cyanobacterium *Synechocystis* sp. PCC 6803 inferred from proteomics and multivariate sequence analyses. J Proteome Res.

[27] Rowland JG, Simon WJ, Nishiyama Y, Slabas AR (2010). Differential proteomic analysis using iTRAQ reveals changes in thylakoids associated with Photosystem II-acquired thermotolerance in *Synechocystis* sp. PCC 6803. Proteomics.

[28] Brandt U, Kerscher S, Dröse S, Zwicker K, Zickermann V (2003). Proton pumping by NADH: ubiquinone oxidoreductase. A redox driven conformational change mechanism?. FEBS Lett.

[29] Yagi T, Yano T, Di Bernardo S, Matsuno-Yagi A (1998). Procaryotic complex I (NDH-1), an overview. BBA-Bioenerg.

[30] Battchikova N, Aro EM (2007). Cyanobacterial NDH-1 complexes: multiplicity in function and subunit composition. Physiol Plant.

[31] Schmetterer G, Alge D, Gregor W (1994). Deletion of cytochrome c oxidase genes from the cyanobacterium *Synechocystis* sp. PCC 6803: evidence for alternative respiratory pathways. Photosynth Res.

[32] Mikami K, Kanesaki Y, Suzuki I, Murata N (2002). The histidine kinase Hik33 perceives osmotic stress and cold stress in *Synechocystis* sp. PCC 6803. Mol Microbiol.

[33] Tabei Y, Okada K, Tsuzuki M (2007). Sll1330 controls the expression of glycolytic genes in *Synechocystis* sp. PCC 6803. Biochem Biophys Res Commun.

[34] Bellou S, Aggelis G (2013). Biochemical activities in *Chlorella* sp. and Nannochloropsis salina during lipid and sugar synthesis in a lab-scale open pond simulating reactor. J Biotechnol.

[35] Anderson PM, Sung Y, Fuchs JA (1990). The cyanase operon and cyanate metabolism. FEMS Microbiol Lett.

[36] Miller AG, Espie GS (1994). Photosynthetic metabolism of cyanate by the cyanobacterium Synechococcus UTEX 625. Arch Microbiol.

[37] Kurian D, Phadwal K, Maenpaa P (2006). Proteomic characterization of acid stress response in *Synechocystis* sp. PCC 6803. Proteomics.

[38] Pitt FD, Mazard S, Humphreys L, Scanlan DJ (2010). Functional characterization of *Synechocystis* sp. strain PCC 6803 pst1 and pst2 gene clusters reveals a novel strategy for phosphate uptake in a freshwater cyanobacterium. J Bacteriol.

[39] Mikami K, Suzuki I, Murata N (2004). Sensors of abiotic stress in Synechocystis. Plant Responses Abiot Stress.

[40] Kashino Y, Lauber WM, Carroll JA, Wang Q, Whitmarsh J, Satoh K (2002). Proteomic analysis of a highly active photosystem II preparation from the cyanobacterium *Synechocystis* sp. PCC 6803 reveals the presence of novel polypeptides. Biochemistry.

[41] Kobayashi M, Shimizu S (1999). Cobalt proteins. Eur J Biochem.

[42] Huertas MJ, López-Maury L, Giner-Lamia J, Sánchez-Riego AM, Florencio FJ (2014). Metals in cyanobacteria: analysis of the copper, nickel, cobalt and arsenic homeostasis mechanisms. Life.

[43] Prakash JS, Sinetova M, Zorina A, Kupriyanova E, Suzuki I, Murata N (2009). DNA supercoiling regulates the stress-inducible expression of genes in the cyanobacterium Synechocystis. Mol BioSyst.

[44] Detmers FJ, Lanfermeijer FC, Poolman B (2001). Peptides and ATP binding cassette peptide transporters. Res Microbiol.

[45] Dunny GM, Leonard BA (1997). Cell-cell communication in gram-positive bacteria. Ann Rev Microbiol.

[46] Lee D-G, Kwon J, Eom C-Y, Kang Y-M, Roh SW, Lee K-B (2015). Directed analysis of cyanobacterial membrane phosphoproteome using stained phosphoproteins and titanium-enriched phosphopeptides. J Microbiol.

[47] Marin K, Suzuki I, Yamaguchi K, Ribbeck K, Yamamoto H, Kanesaki Y (2003). Identification of histidine kinases that act as sensors in the perception of salt stress in *Synechocystis* sp. PCC 6803. Pro Natl Aca Sci USA.

[48] Behshad E, Parkin SE, Bollinger JM (2004). Mechanism of cysteine desulfurase Slr0387 from *Synechocystis* sp. PCC 6803: kinetic analysis of cleavage of the persulfide intermediate by chemical reductants. Biochemistry.

[49] Chen L, Zhu Y, Song Z, Wang J, Zhang W (2014). An orphan response regulator Sll0649 involved in cadmium tolerance and metal homeostasis in photosynthetic *Synechocystis* sp. PCC 6803. J Proteom.

[50] Kamen B (1997). Folate and antifolate pharmacology. Semin Oncol.

[51] Johnson TW, Naithani S, Stewart C, Zybailov B, Jones AD, Golbeck JH (2003). The menD and menE homologs code for 2-succinyl-6-hydroxyl-2, 4-cyclohexadiene-1-carboxylate synthase and *O*-succinylbenzoic acid–CoA synthase in the phylloquinone biosynthetic pathway of *Synechocystis* sp. PCC 6803. BBA-Bioenerg.

[52] Liu M, Han X, Xian M, Ding Y, Liu H, Zhao G (2016). Development of a 3-hydroxypropionate resistant *Escherichia coli* strain. Bioengineered.

[53] Raengpradub S, Wiedmann M, Boor KJ (2008). Comparative analysis of the σB-dependent stress responses in *Listeria monocytogenes* and *Listeria innocua* strains exposed to selected stress conditions. Appl Environ Microb.

[54] Yoshihara S, Geng X, Ikeuchi M (2002). pilG gene cluster and split pilL genes involved in pilus biogenesis, motility and genetic transformation in the cyanobacterium *Synechocystis* sp. PCC 6803. Plant Cell Physiol.

[55] White DJ, Hartzell PL (2000). AglU, a protein required for gliding motility and spore maturation of *Myxococcus xanthus*, is related to WD-repeat proteins. Mol Microbiol.

[56] Dunlop MJ (2011). Engineering microbes for tolerance to next-generation biofuels. Biotechnol Biofuels.

[57] Zheng Y-N, Li L-Z, Xian M, Ma Y-J, Yang J-M, Xu X (2009). Problems with the microbial production of butanol. J Ind Microbiol Biot.

[58] Fang F, Barnum SR (2003). The heat shock gene, htpG, and thermotolerance in the cyanobacterium, *Synechocystis* sp. PCC 6803. Curr Microbiol.

[59] Balogi Z, Cheregi O, Giese KC, Juhász K, Vierling E, Vass I (2008). A mutant small heat shock protein with increased thylakoid association provides an elevated resistance against UV-B damage in Synechocystis 6803. J Biol Chem.

[60] Prakash JS, Krishna PS, Sirisha K, Kanesaki Y, Suzuki I, Shivaji S (2010). An RNA helicase, CrhR, regulates the low-temperature-inducible expression of heat-shock genes groES, groEL1 and groEL2 in *Synechocystis* sp. PCC 6803. Microbiology.

[61] Han M-J, Yoon SS, Lee SY (2001). Proteome analysis of metabolically engineered *Escherichia coli* producing poly (3-Hydroxybutyrate). J Bacteriol.

[62] Trautwein K, Kühner S, Wöhlbrand L, Halder T, Kuchta K, Steinbüchel A (2008). Solvent stress response of the denitrifying bacterium “Aromatoleum aromaticum” strain EbN1. Appl Environ Microb.

[63] Rutherford BJ, Dahl RH, Price RE, Szmidt HL, Benke PI, Mukhopadhyay A (2010). Functional genomic study of exogenous *n*-butanol stress in *Escherichia coli*. Appl Environ Microbiol.

[64] Kildegaard KR, Jensen NB, Schneider K, Czarnotta E, Özdemir E, Klein T (2016). Engineering and systems-level analysis of *Saccharomyces cerevisiae* for production of 3-hydroxypropionic acid via malonyl-CoA reductase-dependent pathway. Microb Cell Fact.

[65] Mukhopadhyay A (2015). Tolerance engineering in bacteria for the production of advanced biofuels and chemicals. Trends Microbiol.

[66] Foo JL, Jensen HM, Dahl RH, George K, Keasling JD, Lee TS (2014). Improving microbial biogasoline production in *Escherichia coli* using tolerance engineering. MBio.

[67] Vega-Palas M, Flores E, Herrero A (1992). NtcA, a global nitrogen regulator from the cyanobacterium Synechococcus that belongs to the Crp family of bacterial regulators. Mol Microbiol.

[68] Kanesaki Y, Yamamoto H, Paithoonrangsarid K, Shumskaya M, Suzuki I, Hayashi H (2007). Histidine kinases play important roles in the perception and signal transduction of hydrogen peroxide in the cyanobacterium, *Synechocystis* sp. PCC 6803. Plant J.

[69] Reumann S, Keegstra K (1999). The endosymbiotic origin of the protein import machinery of chloroplastic envelope membranes. Trends Plant Sci.

[70] Bölter B, Soll J, Schulz A, Hinnah S, Wagner R (1998). Origin of a chloroplast protein importer. Proc Natl Acad Sci.

[71] Liu ZX, Li HC, Wei YP, Chu WY, Chong YL, Long XH (2015). Signal transduction pathways in *Synechocystis* sp. PCC 6803 and biotechnological implications under abiotic stress. Crit Rev Biotechnol.

[72] Rippka R, Deruelles J, Waterbury JB, Herdman M, Stanier RY (1979). Generic assignments, strain histories and properties of pure cultures of cyanobacteria. J Gen Microbiol.

[73] Tan X, Yao L, Gao Q, Wang W, Qi F, Lu X (2011). Photosynthesis driven conversion of carbon dioxide to fatty alcohols and hydrocarbons in cyanobacteria. Metab Eng.

[74] Bradford MM (1976). A rapid and sensitive method for the quantitation of microgram quantities of protein utilizing the principle of protein-dye binding. Analy Biochem.

[75] Vizcaíno JA, Csordas A, del-Toro N, Dianes JA, Griss J, Lavidas I, Mayer G, Perez-Riverol Y, Reisinger F, Ternent T, Xu QW, Wang R, Hermjakob H (2016). 2016 update of the PRIDE database and related tools. Nucl Acids Res.

[76] Zhao P, Liu P, Shao J, Li C, Wang B, Guo X, Yan B, Xia Y, Peng M (2014). Analysis of different strategies adapted by two cassava cultivars in response to drought stress: ensuring survival or continuing growth. J Exp Bot.

[77] Wang Y, Shi M, Niu X, Zhang X, Gao L, Chen L (2014). Metabolomic basis of laboratory evolution of butanol tolerance in photosynthetic *Synechocystis* sp. PCC 6803. Microb Cell Fact.

[78] Ledauphin M, Lemilbeau C, Barillier D, Hennequin D (2010). Differences in the volatile compositions of French labeled brandies (Armagnac, Calvados, Cognac, and Mirabelle) using GC-MS and PLS-DA. J Agr Food Chem.

[79] Liu X, Sheng J, Curtiss R (2011). Fatty acid production in genetically modified cyanobacteria. Proc Natl Acad Sci USA.

[80] Gao W, Zhang W, Meldrum DR (2011). RT-qPCR based quantitative analysis of gene expression in single bacterial cells. J Microbiol Method.

[81] Livak KJ, Schmittgen TD (2001). Analysis of relative gene expression data using real-time quantitative PCR and the 2^−ΔΔCT^ method. Methods.

