# Supplementary material for: Proteomic and metabolomic analyses reveal metabolic responses to 3-hydroxypropionic acid synthesized internally in cyanobacterium Synechocystis sp. PCC 6803
Source: Biotechnol Biofuels. 2016 Oct 6;9:209. doi: 10.1186/s13068-016-0627-6 (PMC5053081; doi:10.1186/s13068-016-0627-6)
Supplement: Supplementary file 1 — 10.1186/s13068-016-0627-6 Comparison of cell growth of the WT and the engineered Synechocystis strains in this study. [file 13068_2016_627_MOESM1_ESM.ppt]

## Slide 1
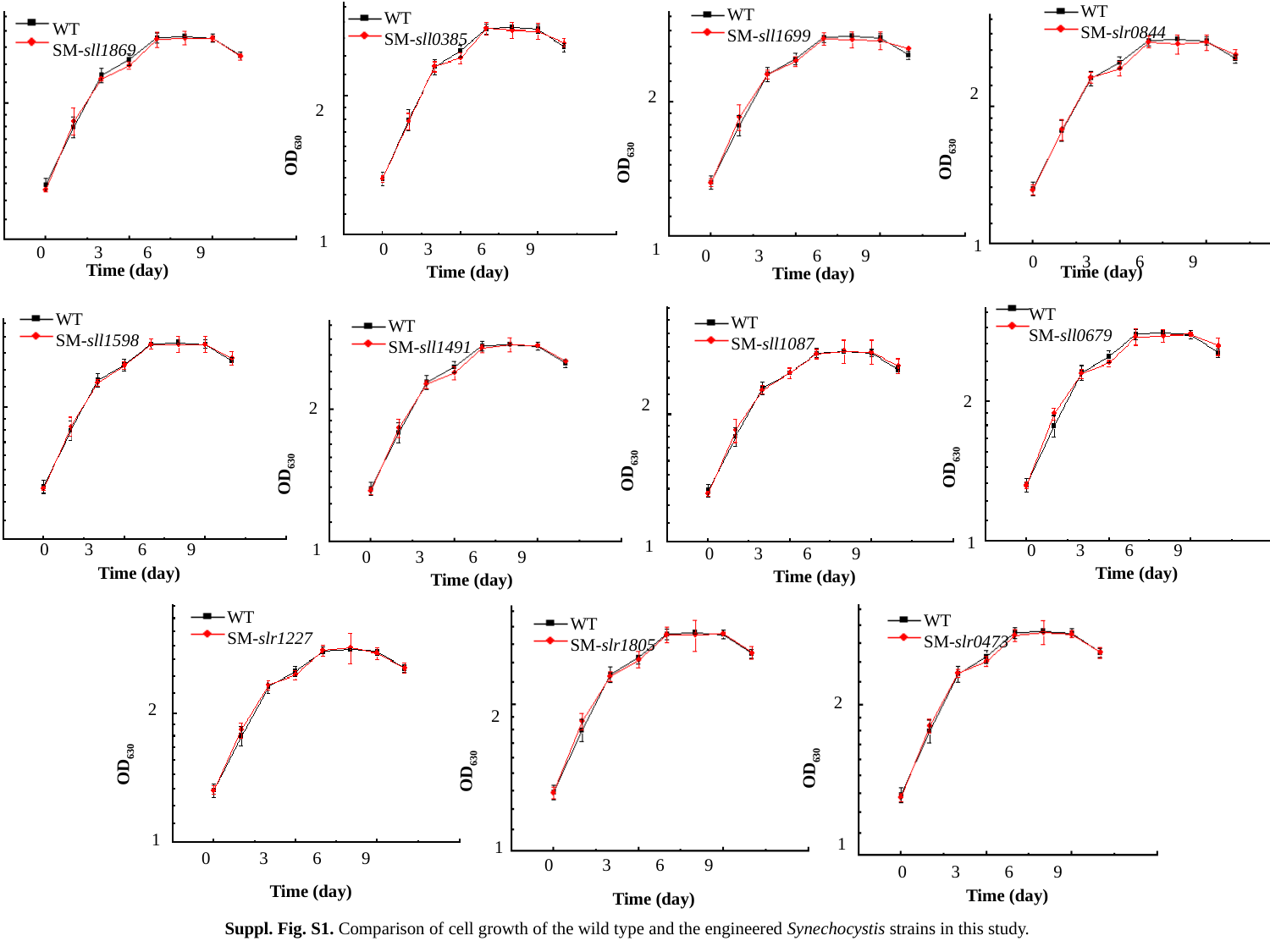

WT
SM-slr0844
WT
SM-sll1699
WT
SM-sll0385
WT
SM-sll1869
2
2
2
2
OD630
OD630
OD630
OD630
1
1
1
1
0 3 6 9
0 3 6 9
0 3 6 9
0 3 6 9
Time (day)
Time (day)
Time (day)
Time (day)
WT
SM-sll0679
WT
SM-sll1598
WT
SM-sll1087
WT
SM-sll1491
2
2
2
2
OD630
OD630
OD630
OD630
1
1
1
0 3 6 9
1
0 3 6 9
0 3 6 9
0 3 6 9
Time (day)
Time (day)
Time (day)
Time (day)
WT
SM-slr1227
WT
SM-slr0473
WT
SM-slr1805
2
2
2
OD630
OD630
OD630
1
1
1
0 3 6 9
0 3 6 9
0 3 6 9
Time (day)
Time (day)
Time (day)
Suppl. Fig. S1. Comparison of cell growth of the wild type and the engineered Synechocystis strains in this study.
